# Supplementary material for: A transient mutational burst occurs during yeast colony development
Source: Mol Syst Biol. 2025 Jun 9;21(9):1214–36. doi: 10.1038/s44320-025-00117-1 (PMC12405527; doi:10.1038/s44320-025-00117-1)
Supplement: Supplementary file 1 — Table EV1 [file 44320_2025_117_MOESM1_ESM.docx]

# ***Table EV1: Single and double mutation rates estimated from fluctuation tests***

| **Strain_condition** | **Fold-change compared to reference** | **rate (mutation/cell/division)** | **number of independent cultures** |
| --- | --- | --- | --- |
| Duplication | | | |
| Reference_colony | 1 | 3.3x10^-6^ (2.7x10^-6^ ; 3.9x10^-6^)  *2.2x10^-6^ (1.7x10^-6^ ; 2.8x10^-6^)* | 63 |
| Reference_colony* | 1 | 6.7x10^-6^ (5.6x10^-6^ ; 7.8x10^-6^)  *4x10^-6^ (3.1x10^-6^ ; 5x10^-6^)* | 107 |
| Reference_liquid | 1.4 | 4.7x10^-6^ (4.3x10^-6^ ; 5.1x10^-6^)  *2.6x10^-6^ (2.3x10^-6^ ; 2.9x10^-6^)* | 90 |
| Reference+HU | 3.6 | 1.2x10^-5^ (1.1x10^-5^ ; 1.3x10^-5^)  *5.5x10^-6^ (4.6x10^-6^ ; 6.6x10^-6^)* | 30 |
| *rad27Δ* | 2.9 | 9.7x10^-6^ (8.7x10^-6^ ; 1.1x10^-5^)  *4.5x10^-6^ (3.7x10^-6^ ; 5.3x10^-6^)* | 30 |
| *rad18Δ* | 1.5 | 4.9x10^-6^ (4.5x10^-6^ ; 5.4x10^-6^)  *2.5x10^-6^ (2.1x10^-6^ ; 3x10^-6^)* | 60 |
| *rad5Δ* | 1.6 | 5.2x10^-6^ (4.7x10^-6^ ; 5.7x10^-6^)  *2.6x10^-6^ (2.2x10^-6^ ; 3x10^-6^)* | 58 |
| *srs2Δ* | 1.8 | 6x10^-6^ (5.5x10^-6^ ; 6.5x10^-6^)  *3x10^-6^ (2.6x10^-6^ ; 3.5x10^-6^)* | 60 |
| *elg1Δ* | 2.2 | 7.3x10^-6^ (6.5x10^-6^ ; 8x10^-6^)  *3.3x10^-6^ (2.7x10^-6^ ; 4x10^-6^)* | 30 |
| *rad52Δ** | < 0.1 | 2.3x10^-7^ (1.6x10^-7^ ; 3x10^-7^)  *1.7x10^-7^ (1x10^-7^ ; 2.4x10^-7^)* | 28 |
| reference_T* | 0.9 | 5.8x10^-6^ (4.8x10^-6^ ; 6.8x10^-6^)  *3.5x10^-6^ (2.6x10^-6^ ; 4.5x10^-6^)* | 49 |
| reference_C | 0.6 | 1.8x10^-6^ (1.2x10^-6^ ; 2.6x10^-6^)  *1.3x10^-6^ (7.4x10^-7^ ; 2x10^-6^)* | 30 |
|  |  |  |  |
|  |  |  |  |
|  |  |  |  |
| **Strain_condition** | **Fold change compared to reference** | **rate (mutation/cell/division)** | **number of independent cultures** |
| Canavanine resistance | | | |
| Reference_colony | 1 | 1.6x10^-7^ (1.5x10^-7^ ; 1.8x10^-7^)  *1.4x10^-7^ (1.2x10^-7^ ; 1.5x10^-7^)* | 104 |
| Reference_liquid | 1.2 | 1.9x10^-7^ (1.7x10^-7^ ; 2.1x10^-7^)  *1.3x10^-7^ (1.1x10^-7^ ; 1.6x10^-7^)* | 90 |
| Reference+HU | 2.8 | 4.4x10^-7^ (3.6x10^-7^ ; 5.2x10^-7^)  *2.9x10^-7^ (2.2x10^-7^ ; 3.7x10^-7^)* | 30 |
| *rad27Δ* | 3.1 | 5x10^-6^ (4.3x10^-6^ ; 5.8x10^-6^)  *2.7x10^-6^ (2.1x10^-6^ ; 3.3x10^-6^)* | 30 |
| *rad18Δ* | 4.1 | 6.6x10^-7^ (5.8x10^-7^ ; 7.4x10^-7^)  *4.3x10^-7^ (5.1x10^-7^ ; 3.6x10^-7^)* | 60 |
| *rad5Δ* | 5.1 | 8.2x10^-7^ (7.3x10^-7^ ; 9.1x10^-7^)  *5.2x10^-7^ (4.5x10^-7^ ; 6x10^-7^)* | 60 |
| *srs2Δ* | 0.7 | 1.1x10^-7^ (8.6x10^-8^ ; 1.3x10^-7^)  8.5x10^-8^ (6.4x10^-8^ ; 1.1x10^-7^) | 60 |
| *elg1Δ* | 3 | 4.8x10^-7^ (4.1x10^-7^ ; 5.6x10^-7^)  *3x10^-7^ (2.4x10^-7^ ; 3.7x10^-7^)* | 60 |
| reference_D | 1.7 | 2.7x10^-7^ (2.3x10^-7^ ; 3x10^-7^)  *2.2x10^-7^ (1.9x10^-7^ ; 2.6x10^-7^)* | 30 |
| *DT1* | 1.2 | 1.9x10^-7^ (1.6x10^-7^ ; 2.1x10^-7^)  *1.6x10^-7^ (1.3x10^-7^ ; 1.8x10^-7^)* | 35 |
| *DT2* | 0.6 | 1x10^-7^ (8.3x10^-8^ ; 1.2x10^-7^)  *8.6x10^-8^ (7.1x10^-8^ ; 1x10^-7^)* | 35 |
|  |  |  |  |
|  |  |  |  |
|  |  |  |  |
|  |  |  |  |
|  |  |  |  |
| **Strain_condition** | **Fold change compared to reference** | **rate (mutation/cell/division)** | **number of independent cultures** |
| Translocation | | | |
| Reference_colony | 1 | 1.8x10^-8^ (1.6x10^-8^ ; 2.1x10^-8^)  *1.7x10^-8^ (1.4x10^-8^ ; 2x10^-8^)* | 229 |
| *rad52Δ* | na | No mutant obtained | 35 |
| reference_D | 1 | 1.8x10^-8^ (1.4x10^-8^ ; 2.3x10^-8^)  *1.7x10^-8^ (1.3x10^-8^ ; 2.2x10^-8^)* | 49 |
| *DC1* | 0.9 | 1.6x10^-8^ (1.2x10^-8^ ; 2.2x10^-8^)  *1.5x10^-8^ (1x10^-8^ ; 2.1x10^-8^)* | 32 |
| *DC2* | 0.8 | 1.5x10^-8^ (1.1x10^-8^ ; 2x10^-8^)  *1.4x10^-8^ (9.8x10^-9^ ; 2x10^-8^)* | 37 |
| *DC3* | 0.9 | 1.7x10^-8^ (1.2x10^-8^ ; 2.3x10^-8^)  *1.6x10^-8^ (1x10^-8^ ; 2.3x10^-8^)* | 30 |
| *DC4* | 1.1 | 1.9x10^-8^ (1.4x10^-8^ ; 2.6x10^-8^)  *1.8x10^-8^ (1.2x10^-8^ ; 2.5x10^-8^)* | 30 |
| *DC5* | 0.8 | 1.5x10^-8^ (1.1x10^-8^ ; 2x10^-8^)  *1.4x10^-8^ (9.2x10^-9^ ; 1.9x10^-8^)* | 34 |
| *DC6* | 1.1 | 1.9x10^-8^ (1.4x10^-8^ ; 2.5x10^-8^)  *1.8x10^-8^ (1.2x10^-8^ ; 2.4x10^-8^)* | 38 |
| *DC7* | 1 | 1.8x10^-8^ (1.4x10^-8^ ; 2.3x10^-8^)  *1.7x10^-8^ (1.2x10^-8^ ; 2.2x10^-8^)* | 38 |
| *DC8* | 0.7 | 1.3x10^-8^ (8.1x10^-9^ ; 1.9x10^-8^)  *1.3x10^-8^ (7.4x10^-9^ ; 1.9x10^-8^)* | 36 |
| *DC9* | 1.2 | 2.2x10^-8^ (1.6x10^-8^ ; 2.8x10^-8^)  *2x10^-8^ (1.4x10^-8^ ; 2.7x10^-8^)* | 30 |
| *DC10* | 1.6 | 2.8x10^-8^ (2.1x10^-8^ ; 3.6x10^-8^)  *2.6x10^-8^ (1.8x10^-8^ ; 3.4x10^-8^)* | 30 |
| *DC11* | 1.4 | 2.5x10^-8^ (1.9x10^-8^ ; 3.2x10^-8^)  *2.3x10^-8^ (1.6x10^-8^ ; 3.1x10^-8^)* | 35 |
| *DC12* | 1.4 | 2.6x10^-8^ (1.9x10^-8^ ; 3.3x10^-8^)  *2.4x10^-8^ (1.7x10^-8^ ; 3.1x10^-8^)* | 32 |
| **Strain_condition** | **Fold change compared to reference** | **rate (mutation/cell/division)** | **number of independent cultures** |
| Translocation | | | |
| *DC13* | 1.4 | 2.5x10^-8^ (1.8x10^-8^ ; 3.2x10^-8^)  *2.3x10^-8^ (1.6x10^-8^ ; 3.1x10^-8^)* | 34 |
| *DC14* | 1.5 | 2.7x10^-8^ (2.1x10^-8^ ; 3.5x10^-8^)  *2.5x10^-8^ (1.8x10^-8^ ; 3.4x10^-8^)* | 30 |
| *DC15* | 1.2 | 2.2x10^-8^ (1.7x10^-8^ ; 2.9x10^-8^)  *2.1x10^-8^ (1.5x10^-8^ ; 2.8x10^-8^)* | 35 |
| *DC16* | 1.2 | 2.1x10^-8^ (1.5x10^-8^ ; 2.8x10^-8^)  *2x10^-8^ (1.3x10^-8^ ; 2.7x10^-8^)* | 30 |
|  |  |  |  |
| **Strain_condition** | **Fold change compared to reference** | **rate (mutation/cell/division)** | **number of independent cultures** |
| Duplication and Canavanine resistance | | | |
| Reference_colony | 1 | 1.8x10^-10^ (1.1x10^-10^ ; 2.9x10^-10^)  *1.8x10^-10^ (1.3x10^-10^ ; 2.4x10^-10^)* | 550 |
| Reference_liquid | 0.9 | 1.6x10^-10^ (8.6x10^-11^ ; 2.6x10^-10^)  *1.6x10^-10^ (1x10^-10^ ; 2.2x10^-10^)* | 343 |
| Reference+HU | 12.2 | 2.2x10^-9^ (1.2x10^-9^ ; 3.6x10^-9^)  *2.2x10^-9^ (1.3x10^-9^ ; 3.3x10^-9^)* | 236 |
| *rad27Δ* | 133.3 | 2.4x10^-8^ (1.8x10^-8^ ; 3x10^-8^)  *2.2x10^-8^ (1.6x10^-8^ ; 2.8x10^-8^)* | 50 |
| *rad18Δ* | 5.5 | 8.8x10^-10^ (5.3x10^-10^ ; 1.4x10^-9^)  8.8x10^-10^ (5.7x10^-10^ ; 1.2x10^-9^) | 149 |
| *rad5Δ* | 8.3 | 1.5x10^-9^ (1x10^-9^ ; 2.1x10^-9^)  1.5x10^-9^ (1x10^-9^ ; 2x10^-9^) | 133 |
| *srs2Δ* | 0.2 | 2.8x10^-11^ (4.7x10^-12^ ; 8.7x10^-11^)  *2.8x10^-11^ (1.3x10^-11^ ; 4.7x10^-11^)* | 493 |
| *elg1Δ* | 2.1 | 3.8x10^-10^ (1.9x10^-10^ ; 6.7x10^-10^)  *3.8x10^-10^ (2.2x10^-10^ ; 2.7x10^-10^)* | 167 |
|  |  |  |  |
| **Strain_condition** | **Fold change compared to reference** | **rate (mutation/cell/division)** | **number of independent cultures** |
| Duplication and Translocation | | | |
| Reference_colony | 1 | 1.1x10^-11^ (1.8x10^-12^ ; 3.4x10^-11^)  *1.1x10^-11^ (5.9x10^-12^ ; 1.7x10^-11^)* | 1006 |

Rates measured with R-Salvador are in regular text while rates measured with FluCalc are italicized. * Duplication rates were measured from dropout synthetic media from Sigma (see Material and Method).

# 
