# Supplementary material for: A transient mutational burst occurs during yeast colony development
Source: Mol Syst Biol. 2025 Jun 9;21(9):1214–36. doi: 10.1038/s44320-025-00117-1 (PMC12405527; doi:10.1038/s44320-025-00117-1)
Supplement: Supplementary file 2 — Table EV2 [file 44320_2025_117_MOESM2_ESM.docx]

# ***Table EV2: Comparison of the distributions of double mutants between simulations and experimental observations***

| **Mutator Duration**  **(in generation)** | **Mutator population size** | **Mutator increase fold change** | ***p***  **obs vs simu** |
| --- | --- | --- | --- |
| 1 | 10 | 10000 | 0.08 |
| 1 | 50 | 4000 | 0.99 |
| 1 | 75 | 4000 | 0.28 |
| 1 | 100 | 4000 | 0.7 |
| 1 | 250 | 3000 | 0.04 |
| 1 | 500 | 2300 | 0.91 |
| 1 | 1000 | 1800 | 0.7 |
| 5 | 10 | 2000 | 0.04 |
| 5 | 100 | 400 | 0.47 |
| 5 | 100 | 600 | 0.7 |

P*-value* (*p*) were computed using Kolmogorov-Smirnov tests. The combinations of mutator duration, population size and fold increase are compatible with our experimentally observed single *D* and *C* and double  *DC* mutation rate (see green symbols on Fig 2).
