# Supplementary material for: A transient mutational burst occurs during yeast colony development
Source: Mol Syst Biol. 2025 Jun 9;21(9):1214–36. doi: 10.1038/s44320-025-00117-1 (PMC12405527; doi:10.1038/s44320-025-00117-1)
Supplement: Supplementary file 3 — Table EV3 [file 44320_2025_117_MOESM3_ESM.docx]

# ***Table EV3: Non-selected additional mutations in DC double mutants***

| **Strain** | **Mutation** | **chromosome (position)** |
| --- | --- | --- |
| *DC2* | C->T | chr. II (543543) |
| *DC2* | A->G | chr. IX (196173) |
| *DC3* | C->T | chr. IV (317700) |
| *DC3* | A->T | chr. VII (93938) |
| *DC3* | C->T | chr. VIII (27534) |
| *DC3* | C->T | chr. VIII (357057) |
| *DC3* | A->C | chr. XII (916399) |
| *DC3* | A->G | chr. XIII (758436) |
| *DC3* | G->T | chr. XIV (49294) |
| *DC3* | G->A | chr. XIV (505623) |
| *DC3* | -1A | chr. XVI (324182) |
| *DC3* | -1T | chr. XVI (630607) |
| *DC3* | T->A | chr. XVI (630611) |
| *DC8* | G->A | chr. IV (1503778) |
| *DC8* | G->C | chr. XIV (188270) |
| *DC11* | A->T | chr. XV (47796) |
| *DC12* | A->C | chr. XII (265263) |
| *DC13* | T->C | chr.XI (334602) |
| *DC13* | A->C | chr. XIII (352923) |
| *DC17* | C->T | chr.XV (511471) |
| *DC22* | A->T | chr. X (511046) |
| *DC23* | G->T | chr. IX (312169) |
| *DC24* | C->A | chr. XVI (872400) |
| *DC25* | G->A | chr. I (25488) |
| *DC26* | G->C | chr. X (283044) |
| *DC27* | C->A | chr. I (86059) |
| *DC30b* | A->C | chr. XV (85190) |

Mutations were identified by Illumina sequencing of the 35 *DC* double mutants. Coordinates refer to the reference genome sequence (*S288C_reference_sequence_R64-4-1_20230830*). Mutations detected in repeated sequences such as telomeres or LTRs were filtered out.
