# Supplementary material for: A transient mutational burst occurs during yeast colony development
Source: Mol Syst Biol. 2025 Jun 9;21(9):1214–36. doi: 10.1038/s44320-025-00117-1 (PMC12405527; doi:10.1038/s44320-025-00117-1)
Supplement: Supplementary file 4 — Table EV4 [file 44320_2025_117_MOESM4_ESM.docx]

# ***Table EV4: Strains table***

| **Strain name** | **Genotype** |
| --- | --- |
| YAG142 | YMR242c, next s*igma3::URA3delta5'short::HIS3, sigma4::URA3delta3'short::LEU2, his3D1, leu2D0, LYS2, MET15, trp1Δ5'(1-362)::natNT2, CYC1Δ::TRP1Δ3'(long)-hph* |
| YAG163 | YMR242c, next *sigma3::URA3delta5'short::HIS3, sigma4::URA3delta3'short::LEU2, his3D1, leu2D0, LYS2, MET15, TRP1Δ5'(1-362)::natNT2, CYC1Δ::TRP1Δ3'(long)-hph, rad5Δ::KanMX4* |
| YAG164 | YMR242c, next *sigma3::URA3delta5'short::HIS3, sigma4::URA3delta3'short::LEU2, his3D1, leu2D0, LYS2, MET15, TRP1Δ5'(1-362)::natNT2, CYC1Δ::TRP1Δ3'(long)-hph, rad27Δ::KanMX4* |
| YAG165 | YMR242c, next *sigma3::URA3delta5'short::HIS3, sigma4::URA3delta3'short::LEU2, his3D1, leu2D0, LYS2, MET15, TRP1Δ5'(1-362)::natNT2, CYC1Δ::TRP1Δ3'(long)-hph, rad18Δ::KanMX4* |
| YAG166 | YMR242c, next *sigma3::URA3delta5'short::HIS3, sigma4::URA3delta3'short::LEU2, his3D1, leu2D0, LYS2, MET15, TRP1Δ5'(1-362)::natNT2, CYC1Δ::TRP1Δ3'(long)-hph, elg1Δ::KanMX4* |
| YAG169 | YMR242c, next *sigma3::URA3delta5'short::HIS3, sigma4::URA3delta3'short::LEU2, his3D1, leu2D0, LYS2, MET15, TRP1Δ5'(1-362)::natNT2, CYC1Δ::TRP1Δ3'(long)-hph,srs2Δ::KanMX4* |
| YAG182 | YMR242c, next *sigma3::URA3delta5'short::HIS3, sigma4::URA3delta3'short::LEU2, his3D1, leu2D0, LYS2, MET15, trp1Δ5'(1-362)::natNT2, CYC1Δ::TRP1Δ3'(long)-hph, rad52Δ::KanMX4* |
