# Supplementary material for: A transient mutational burst occurs during yeast colony development
Source: Mol Syst Biol. 2025 Jun 9;21(9):1214–36. doi: 10.1038/s44320-025-00117-1 (PMC12405527; doi:10.1038/s44320-025-00117-1)
Supplement: Supplementary file 6 — Expanded View Figures [file 44320_2025_117_MOESM6_ESM.pdf]

Expanded View Figures

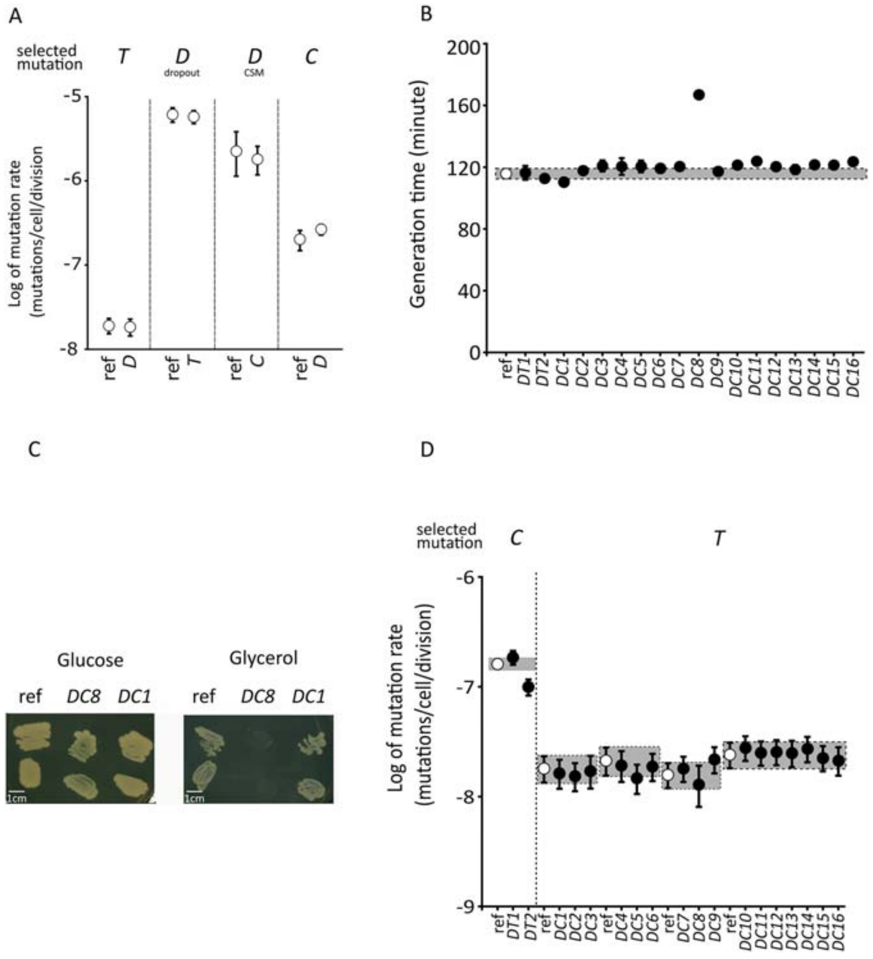

Figure EV1. Properties of the single and double mutants.

(**A**) Comparison of mutation rates between the YAG142 reference strain (ref) and the single mutants carrying the translocation (*T*), the segmental duplication (*D*), or the resistance to canavanine mutation (*C*). The single *T* mutation rate was measured in a *D* mutant ($n = 49$ independent cultures). The single *D* mutation rate was measured in either the *T* ($n = 49$ independent cultures) or *C* ($n = 30$ independent cultures) mutants using two synthetic media, the "dropout" (Sigma-Aldrich) or the CSM (MP Biotech). The single *C* mutation rate was measured in a *D* mutant ($n = 30$ independent cultures). Error bars represent the 95% likelihood ratio confidence intervals. (**B**) The generation time in minute of the YAG142 reference strain (ref - open circle) and the duplication and translocation (*DT*) or Duplication and Canavanine resistant (*DC*) double mutants (black circle), grown in YPD broth at 28 °C without agitation. Each point represents the average of a minimum of three replicates. Error bars represent standard deviation to the mean. (**C**) Cellular patches of the parental strain YAG142 (ref), *DC8* and *DC1* double mutants grown at 30 °C on YP-glucose (a fermentable carbon source) and YP-glycerol (a non-fermentable carbon source). (**D**) Comparison of mutation rates between the YAG142 reference strain (ref, open circles) and the double mutants carrying the duplication and translocation (*DT*) or Duplication and Canavanine resistant (*DC*, black circles). The single *C* and *T* mutation rates were measured in the 2 *DT* and 16 *DC* double mutants, respectively. The *DC* strains were tested in four different batches with each time the reference strain used as control. For each strain, a minimum of 30 independent cultures were used to measure the mutation rate. Error bars represent the 95% likelihood ratio confidence intervals.

 © The Author(s)

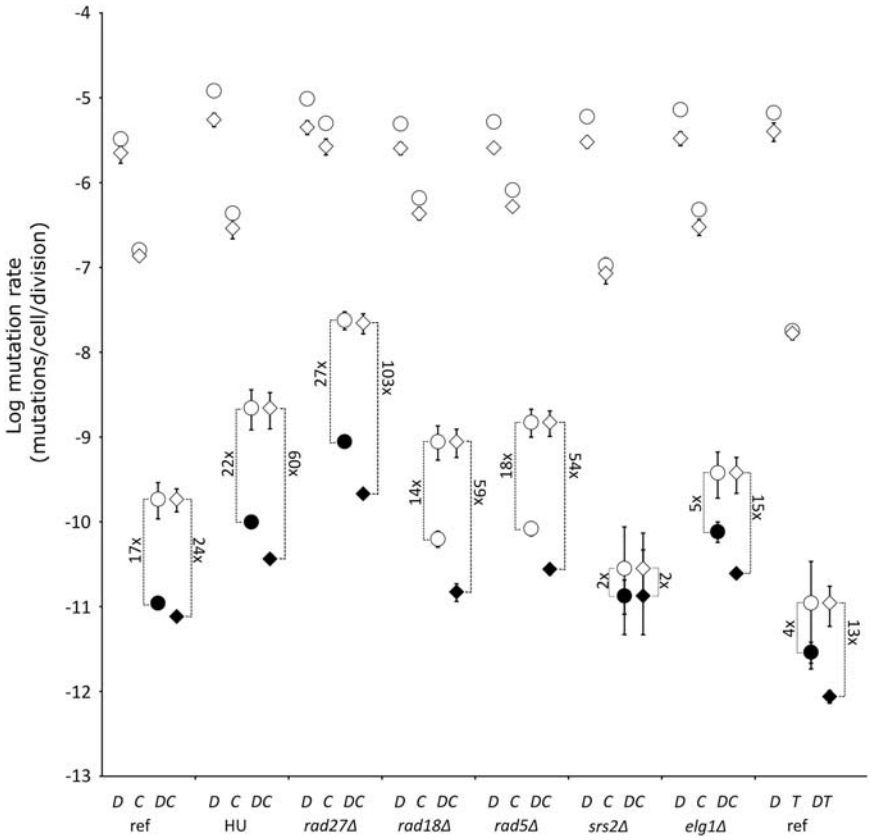

Figure EV2. Comparison of two methods for estimating mutation rates.

The experimentally measured single mutation rates for the segmental duplication (*D*), canavanine resistance (*C*) and reciprocal translocation (*T*), and double mutation rates for duplication and canavanine resistance (*DC*) and duplication and translocation (*DT*) are symbolized by open circles when calculated by the newton.LD.plating function of R-Salvador and open diamond when calculated by the MSS-MLE method with the web application FluCalc. The reference strain YAG142 is noted (ref). The number of independent cultures performed to measure *D* is: ref: 63, HU: 30, *rad27Δ*: 30, *rad18Δ*: 60, *rad5Δ*: 58, *srs2Δ*: 60, *elg1Δ*: 30. The number of independent cultures performed to measure *C* is: ref: 104, HU: 30, *rad27Δ*: 30, *rad18Δ*: 60, *rad5Δ*: 60, *srs2Δ*: 60, *elg1Δ*: 60. The number of independent cultures performed to measure *DC* is: ref: 550, HU: 236, *rad27Δ*: 50, *rad18Δ*: 149, *rad5Δ*: 133, *srs2Δ*: 493, *elg1Δ*: 167. For the reference strain the number of independent cultures performed to measure *T* is 229 and to measure *DT* 1006. The theoretical double mutation rates (*DC*) and (*DT*) estimated with a null model of mutation accumulation are indicated by closed circles or diamonds. For each point at least 1,000 realizations were performed to calculate the theoretical mutation rates. The fold changes between the observed and theoretical double mutation rates are indicated on the side. Error bars represent the 95% likelihood ratio confidence intervals.

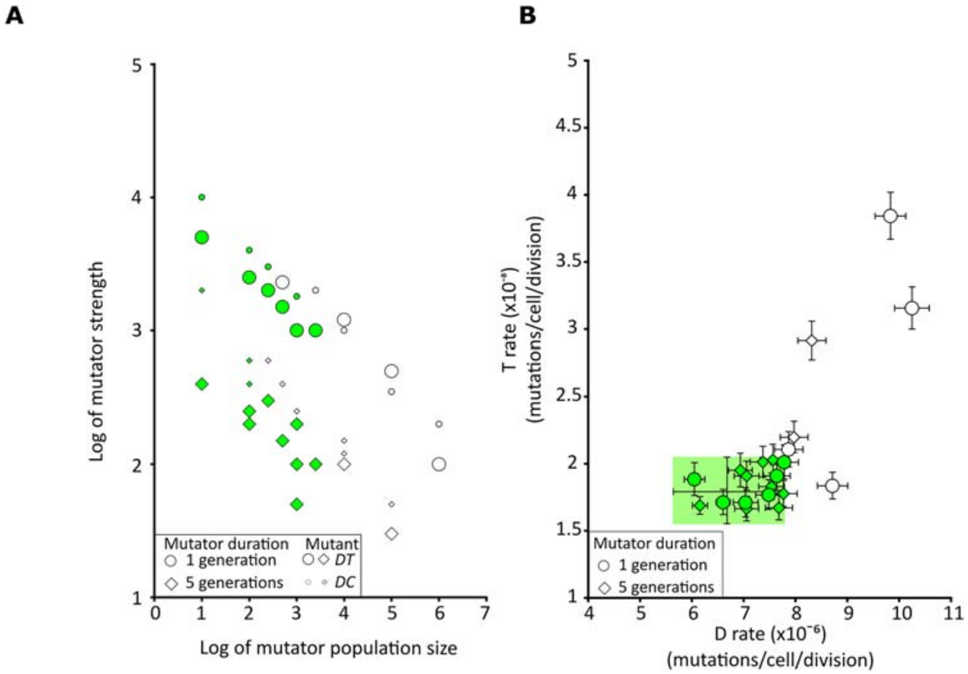

Figure EV3. Exploration of parameter space for the duration, size, and strength of the mutator subpopulation for the formation of DT double mutants.

(**A**) The mutator subpopulation size represents the number of cells that experience the transient mutator phenotype. The mutator strength is expressed as a fold change increase of the general cell mutation rate. The two mutator durations of 1 and 5 generations are symbolized by open circles and diamonds, respectively. The symbol size refers to *DT* (big) and *DC* (small) double mutants. All reported values recapitulate the experimentally observed *DT* and *DC* double mutation rates. For each point at least 1000 realizations of the refined model were performed to calculate the mutation rate. The points shaded in green correspond to combinations of size, strength, and duration of the mutator phenotype that recapitulate the experimentally observed single *D*, *C*, *T*, and double *DC* and *DT* mutation rates as determined by the area shaded in green in the (**B**) panel. (**B**) The symbols are as in the A panel. For each point at least 1000 realizations have been performed to calculate the mutation rates. The area highlighted in green corresponds to the 95% likelihood ratio confidence intervals of the experimentally measured *D* and *T* single mutation rates. The realizations that fall within this area recapitulate the experimental single mutation rates and therefore were colored in green in the (**A**) panel.

 © The Author(s)

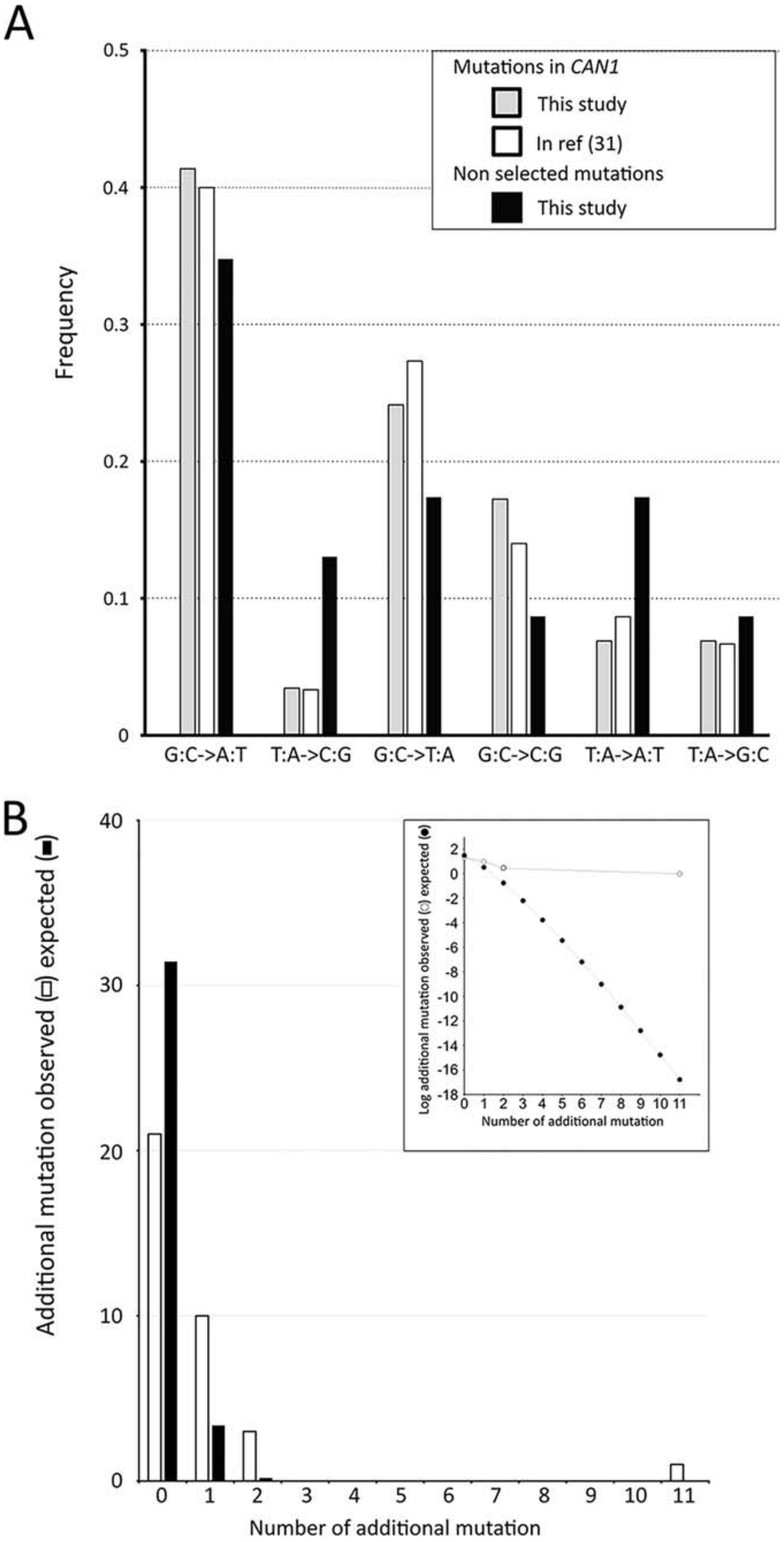
A
Mutations in CAN1
This study
In ref (31)
Non selected mutations
This study
Frequency
0.5
0.4
0.3
0.2
0.1
0
G:C->A:T
T:A->C:G
G:C->T:A
G:C->C:G
T:A->A:T
T:A->G:C
B
Additional mutation observed (□) expected (■)
40
30
20
10
0
0 1 2 3 4 5 6 7 8 9 10 11
Number of additional mutation
Log additional mutation observed (○) expected (●)
Number of additional mutation

Figure EV4. Properties of selected can1 and non-selected additional mutations.

For both panels, the number and type of point mutations identified in this study were determined by whole-genome Illumina sequencing of the 35 *DC* mutants and variant calling using GATK (Auwera and O'Connor, 2020). (**A**) Spectrum of the selected *can1* and non-selected additional point mutations. Selected mutations in the *CAN1* gene identified in this study are symbolized by gray bars and those from reference (Lang and Murray, 2008) by open bars. The non-selected additional mutations identified in this study in the *DC* mutants are represented by black bars. (**B**) Number of observed non-selected additional mutations identified in the 35 *DC* mutants (open bar) and expected additional mutations (black bar). The numbers of expected additional mutations were determined as a random variable following a Binomial distribution, under the hypothesis of a constant mutation rate across cell divisions. The parameters used are: a genome size of 12×10^6 bp, a point substitution rate of 3.3×10^{-10} and a number of cell divisions of 27. Inset: the same data are represented on a log scale. Comparison of the two distributions was performed by Chisquare test giving a p-value $< 2.2 \times 10^{-16}$. The same comparison, excluding the *DC3* mutant and its 11 additional mutations, gave a p-value of 1.2×10^{-9}.

 © The Author(s)

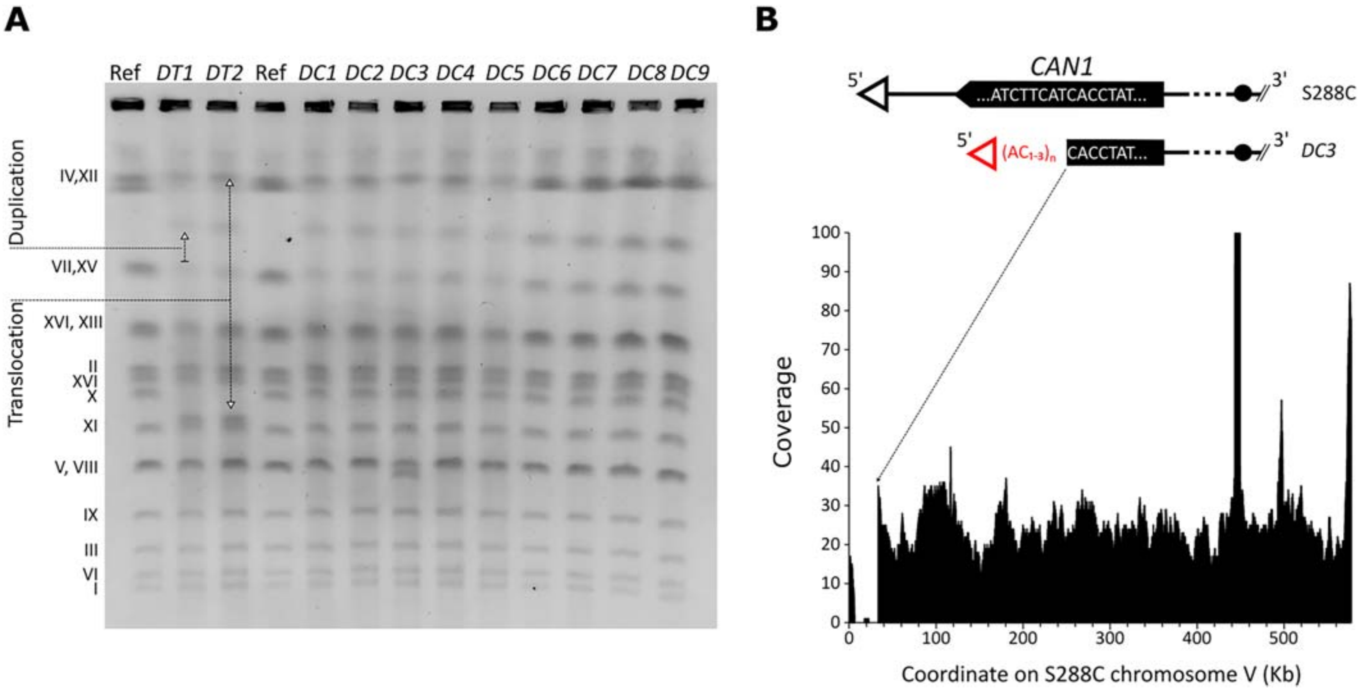

Figure EV5. Characterization of an additional large deletion in the DC3 double mutant.

(**A**) Pulsed-field gel electrophoresis of the parental strain YAG142 (Ref), 2 *DT* and 9 *DC* double mutants. The bands characterizing the selected reciprocal translocation and segmental duplication are indicated by arrows. The *DC3* karyotype shows a smaller band corresponding to either chromosome V or VIII. (**B**) The DNA coverage obtained from de novo genome sequencing along the chromosome V of the *DC3* strain. Upper part shows a schematic of chromosome V left arm in the reference strain S288C and in the *DC3* double mutant at the *CAN1* locus. The deletion occurred within the *CAN1* gene and a new telomere was added using a 5′-GGTG-3′/CACC-3′ seed.
